# Does Ankyloglossia Surgery Promote Normal Facial Development? A Systematic Review

**DOI:** 10.3390/jcm14010081

**Published:** 2024-12-27

**Authors:** Małgorzata Kotarska, Alicja Wądołowska, Michał Sarul, Beata Kawala, Joanna Lis

**Affiliations:** 1 The Department of Integrated Dentistry, Wroclaw Medical University, 50-425 Wroclaw, Poland; michal.sarul@umw.edu.pl; 2 The Department of Dentofacial Orthopaedics and Orthodontic, Wroclaw Medical University, 50-425 Wroclaw, Polandbeata.kawala@umw.edu.pl (B.K.); joanna.lis@umw.edu.pl (J.L.)

**Keywords:** ankyloglossia, tongue-tie, frenotomy, frenulotomy, frenectomy, lower face morphology, interceptive orthodontics

## Abstract

**Background:** Ankyloglossia is a congenital, abnormally short, thickened, or tight lingual frenulum that restricts tongue mobility, which may impair the development of the lower face morphology, namely the occlusion and skeleton. **Objective:** The aim of this study was to evaluate whether and how the lingual frenotomy benefits the occlusion and lower face skeleton development. **Search methods and selection criteria:** The authors, independently and in duplication, performed searches of PubMed, Cochrane Library, Medline, Web of Science, and Embase, introducing the following keywords: tongue tie, ankyloglossia, and short lingual frenum/frenulum, combined with malocclusion, lower face skeleton, and hyoid bone. **Data collection and analysis:** Relevant articles were assessed for quality according to the Cochrane guidelines and the data extracted for further analysis of the risk of bias and the evidence strength. **Results:** Seven articles including 1349 patients with ankyloglossia and 90 in the control group underwent the detailed analysis. The quality of the included studies was assessed as low. The strongest evidence of studies reporting the relationship of ankyloglossia with lower face abnormalities concerns the reduction in the intercanine and intermolar widths in either the maxilla or the mandible, as well as Class III occurrence. **Limitations:** The drawbacks of the analysed papers are mainly composition and number of participants. There is also a lack of good-quality prospective studies, particularly randomised clinical trials, in the literature. **Conclusions:** Although the lack of high-quality studies dictates that we must treat our results cautiously, the gathered evidence conditionally allow us to state the following: 1. ankyloglossia may be one of the factors contributing to maxillary constriction, Class III malocclusion, and mandibular incisor crowding; 2. the patient’s age is relevant when it comes to frenotomy timing. Possible indications for the procedure depend on the patient’s malocclusion.

## 1. Introduction

Ankyloglossia is a congenital, abnormally short, thickened, or tight lingual frenulum that restricts tongue mobility [1]. Estimates range from 2.1% to 10.7% [2], but definitive incidence and prevalence statistics are elusive due to an absence of a standard criterion or diagnostic criteria which could be used in clinical practice [3].

Recently, the annual number of frenotomies have severely increased, although there are no studies reporting a higher incidence of ankyloglossia itself [4,5] and despite the substantial evidence undermining the interference of a tongue-tie with breastfeeding and speech [6,7,8,9,10,11,12,13]. At this point, it should be emphasised that since a standard definition of “interference with breastfeeding” is not available, it leaves room for misinterpretation and only an illusory indication of the need for treatment. The lack of natural history data for untreated ankyloglossia further promulgates uncertainty. Serious complications related to the surgery still remain an important matter and must not be neglected [14,15,16]. Furthermore, some scientists propose that a short frenulum elongates spontaneously due to its thinning with age [17,18]. Altogether, this triggers a question: does one witness an overtreatment that—in the absence of any benefits—only bears the risk of complex, undesirable iatrogenic consequences?

On the other hand, some facts are unquestionable and doubtless: the clinically considerable impact of an improper tongue anatomy on the development of malocclusions such as an open bite or arch length discrepancies [19,20]; not to mention that ankyloglossia co-occurs with improper position of the hyoid bone, which jeopardises airway patency in growing patients [21,22,23,24].

Hence, the re-evaluation of studies dealing with the maxilla and the mandible morphology in patients with ankyloglossia appears to be essential to determine whether that anatomical defect of the tongue impairs its function, thus bearing a risk of malformation of either the lower face skeleton or/and malocclusion. If this is true, it would in turn be recommended to determine a suitable patient’s age for surgery, optimal for either preventing or weakening the symptoms of the above-listed abnormalities.

Thus, the following two null hypotheses have been formulated:

1. A shortened lingual frenulum does not impair the development of the lower face morphology, namely the occlusion and skeleton.
2. The patient’s age is irrelevant when it comes to achieving most of the clinical benefits after lengthening of a short lingual frenulum, the verification of which required the present systematic review.
3. By testing the null hypothesis, we will obtain information whether ankyloglossia affects the lower face morphology and, if so, whether there is an optimal time for the diagnosis or treatment.

## 2. Materials and Methods

The study project was registered at PROSPERO under the number CRD42022333628, in accordance with the Preferred Reporting Items for Systematic Reviews and Meta-Analyses PRISMA 2020 statement guidelines and with use of a PRISMA checklist. The inclusion criteria were determined based on the aspects of Participants, Exposure, Comparison, Outcome, and Study design (PECOS):

Population. Generally healthy patients with no history of lingual frenulum surgery, untreated orthodontically.

Exposure. Patients with shortened lingual frenulum.

Comparison. Patients with normal lingual frenulum length.

Outcome. Abnormalities of the lower face, manifested by malocclusion and/or disturbed skeletal parameters, measured on diagnostic models or scans and in the lateral cephalograms.

Study design. Clinical control trials (CCTs), randomised control trials (RCTs), and cohort studies (CSs) published between 1948 and 09 December 2024.

Studies of patients with congenital abnormalities, animal studies, studies with a study group size of less than 10, and case studies constituted the exclusion criteria.

### 2.1. Search Strategy

**Figure 1:**
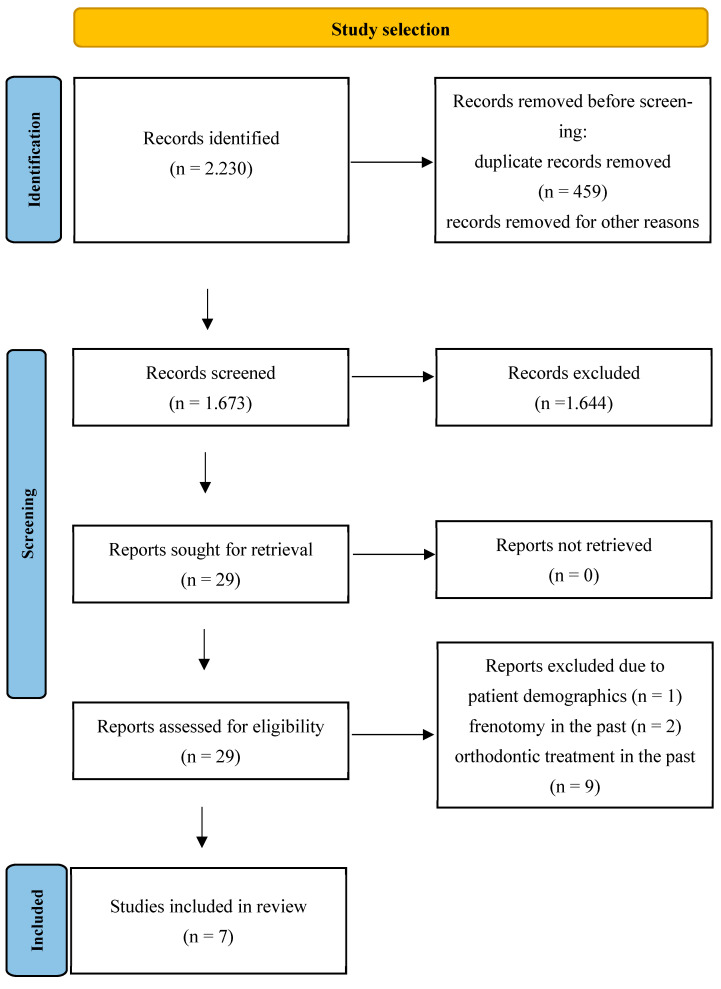
Flow chart demonstrating the study retrieving process.

Publications from the following electronic databases were sources of information: PubMed, Cochrane Library, Medline, Web of Science, and Embase. The key word strategy was as follows: “tongue tie” OR “ankyloglossia” OR “short lingual frenum/frenulum” OR “malocclusion” OR “lower face skeleton” OR “hyoid bone”. The initial selection of articles based on their titles and abstracts, without language restrictions, was aimed at evaluating the papers in terms of whether they met the inclusion criteria. The search and screening processes were conducted simultaneously and independently by two researchers, and the degree of agreement between them was quantified using a κ coefficient of 0.85, indicating a substantial level of concordance. After obtaining full versions of the manuscripts, the same clinicians applied the defined PECOS and exclusion criteria to both the manuscripts and their literature items in order to confirm their suitability for the review. On contentious issues, a third experienced investigator had the deciding vote. The entire selection process is presented in the PRISMA flow chart (Figure 1).

### 2.2. Data Extraction and Quality Assessment Procedures

After selection of the full-text articles, if insufficient data were found, the author/s was/were asked to provide them via e-mail. Failure to respond either entirely excluded the publication from the analysis or lowered the number of points assigned to the publication in further evaluation of the gained evidence.

Finally, seven articles [25,26,27,28,29,30,31] meeting the Cochrane guidelines were qualified and their data were analysed in order to scientifically either confirm or deny two null hypotheses formulated in the introduction of this systematic review. Information about occlusion and the maxillo-facial skeleton parameters obtained from diagnostic models and lateral cephalograms, respectively, together with the details of the tongue-tie evaluation, was collected in a Microsoft Office Excel 2013 spreadsheet (Microsoft Corporation, Redmond, Washington, DC, USA).

### 2.3. Risk of Bias Assessment

To assess the risk of bias, authors used a modified Joanna Briggs Institute (JBI) critical appraisal checklist for the analytical cross-sectional and case-control studies.

### 2.4. Evidence

Two authors independently applied the GRADE method (Grading of Recommendations, Assessment, Development, and Evaluations) to rate the quality of evidence as strong, moderate, low, and very low. When those researchers disagreed, a third investigator was consulted for a discussion with the aim to arrive at a reasonable conclusion.

## 3. Results

The seven studies comprised in the review included 1439 patients, 1349 with ankyloglossia and 90 from control groups.

The pertinent information organised in the distinct sections and the demographic structure of the pooled patient sample extracted from the articles, together with the data subjected to statistical analysis and types of ankyloglossia measurement among studies, are shown in Table 1, Table 2, Table 3, Table 4 and Table 5.

Table 6 comprises the statistical analyses of the median lingual frenulum length (MLFL), mean values, and maximum mouth opening reduction (MMOR) dependent on skeletal classes in the papers by Meenakshi et al. [25] and Jang et al. [26]. Table 7, Table 8 and Table 9 demonstrate comparisons of changes in occlusal and skeletal parameters of the maxillo-facial skeleton in relation to the severity of ankyloglossia in the papers by Jang et al. [26], Srinivasan et al. [27], and Yoon et al. [28], respectively.

Synthesis of the results was impossible due to the small sample sizes, insufficient data provided, or discrepancies between the results in the analysed papers.

### 3.1. General Characteristics of the Included Studies

#### Risk of Bias Assessment

The results of the bias analysis in two CCTs and five CSs are presented in Table 10.

### 3.2. Evidence-Based Results of Individual Studies

Table 11 comprises all the parameters whose disturbance or the occurrence have a statistically significant correlation with ankyloglossia, indicating (a) the evidence strength of the results, (b) information on the increase or decrease in the parameter value in the individual studies. The strongest evidence of studies reporting the relationship of ankyloglossia with lower face abnormalities concerns the reduction in the intercanine and intermolar widths in either the maxilla or the mandible [27,28], as well as Class III occurrence [25,26,28,31]. Unfortunately, all patients involved in the presented studies were recruited from individuals seeking orthodontic treatment, and the selection of the control group was presented only in the article by Srinivasan et al. [27].

The link between ankyloglossia and 1. maxillary constriction, 2. increase in (a) lower incisor irregularity, (b) Mx crowding, (c) palatal slope, (d) overbite, (e) PNS-P distance, (f) APDI distance, (g) L1-NB distance, (h) interincisal angle, (i) SNB angle, and (j) Co-Pg distance, as well as 3. decrease in (a) Mn C W, (b) Go-Gn-SN angle, (c) ANB angle, and (d) WITS appraisal was admittedly confirmed in this review, but the evidence strength of the links was determined to be low, inter alia due to the presentation of evidence in only one study [26,27,28,31], the selection of patients from among those presenting for orthodontic treatment [25,26,27,28,31], conflicting results [27,28,29], or the lack of a control group [25,26,28,30,31].

The relationship of ankyloglossia and disorders such as too-high position of the hyoid bone, anterior open bite, posterior crossbite, deep bite, and posterior crossbite in patients with ankyloglossia was conditionally confirmed due to the very low evidence strength.

### 3.3. Overall Evidence

Since an overall GRADE quality rating can be applied to a body of evidence across outcomes, usually by removing evidence of the lowest quality from all of the outcomes that are critical to decision making, we assessed our evidence to be of low quality.

## 4. Discussion

In 2019, a team of experts consisting of paediatric otolaryngologists undertook a summary of clinical guidelines on the topic of ankyloglossia among children [32]. Since the effect of ankyloglossia on nutrition and proper pronunciation is negligible according to reliable research [6,7,8,9,10,11,12,13], possible indications for a frenotomy should be based on the manner in which the lingual frenulum length affects the lower face morphology. According to Moss’s matrix theory, soft tissue such as the tongue influences the formation of bone structures [33], but the analysis of this phenomenon is hampered by the under-researched association of ankyloglossia with incorrect tongue rest posture [34]. However, the results obtained in our review revealed different individual occlusal and skeletal parameters in patients with ankyloglossia depending on its severity and in comparison to patients without this pathology. Altogether, this allowed us to determine the clinically visible impact of a shortened lingual frenulum on the lower face morphology, despite the demonstrated low value of the evidence.

As far as methodology—namely, the diagnosis of ankyloglossia—is concerned, the literature lists a variety of tools: the Marchesan functional assessment, the questionnaire assessment (particularly useful in the examination of neonates and infants) according to the Alison Halzelbaker scale, and others, such as the assessment criteria according to Corylossa, Ruffoli, Hogan, Ricke, Griffits, Ballad, Messner, Masaitis, Harris, and Market [26,35,36,37,38]. In the papers analysed in this review, both the length of the free tongue part [27,28,29,30] and the tongue function were determined [25,26,28]. In order to assess the anatomy, the Median Lingual Frenulum Length (MLFL) measurement (intra-orally or on the impressions) or Kotlow’s method [18] were applied. In Kotlow’s method, the distance from the apex of the tongue to the attachment of its frenulum (in mm) is measured, which allows us to classify the possible ankyloglossia as follows: 12 to 16—grade 1 (mild); 8 to 11—grade 2 (moderate); 3 to 7—grade 3 (severe); <3—grade 4 (complete ankyloglossia). The tongue function was assessed using Maximum Mouth Opening Reduction (MMOR) or Tongue Range of Motion Ratio (TRMR), with the tongue mobility classified as follows: grade 1 > 80% (complete), grade 2 = 50%–80% (average to mildly restricted), grade 3 < 25% (severely restricted). While Kotlow’s method and the MMOR functional assessment are considered reliable diagnostic tools, the MLFL technique used in the studies by Meenakshi et al. [25] and Jang et al. [26] is questionable, because there is a risk of a measurement error due to the soft tissue compression—with a ruler or impression mass; in addition, this method does not take into account the influence of the length of the free tongue part on the dental and skeletal parameters of the lower face.

In the analysed studies, some authors grouped their patients depending on the severity of ankyloglossia and either investigated the occlusal and skeletal parameters [26,27,30] or determined the occurrence and percentage distribution of distinctive malocclusions [27,30,31], whilst others focused on malocclusions only, without linking them to the severity of ankyloglossia [25,26,28,29].

Assigning all patients with ankyloglossia to one group does not have adverse consequences on the assessment of the maxilla, because—as this systematic review demonstrated—irrespectively of the severity of the lingual frenulum anomaly, the maxillary transverse dimensions measured at the canine (Mx C W) and the molar (Mx M W) levels are always reduced in patients with a tongue-tie.

However, such composition of a study group is a serious drawback of any research which involves assessment of parameters describing the morphology and position of the mandible, which vary considerably depending on the degree of the tongue-tie. While comparing the study group and the control group (without ankyloglossia), Srinivasan et al. [27] found only insignificant differences: a smaller Go-Gn-Sn value and a larger overbite in the control group. However, the authors proved a significant negative correlation between the Go-Gn-Sn value and the grade of ankyloglossia. In this way, they demonstrated that the more severe the ankyloglossia, the greater the anteriorotation of the mandible, which in turn is likely to develop into Class III. Further findings, such as a statistically significant increase in the overbite and decreased crowding of the mandibular incisors, along with the severity of ankyloglossia, clearly indicate the dominance of the sagittal component of the maxillary growth over the transverse one, as well as the forwards growth of the mandible. This is consistent with the results of Sepet et al. [30] and Vaz et al. [31], who observed a significant positive correlation of mild and moderate ankyloglossia with the reduction in mandibular incisor crowding. Once again, this proves that with the shortening of the lingual frenum, the growth of the mandibular alveolar part increases, especially its frontal area. This is regardless of the fact that complete ankyloglossia coexists with an increase in the irregularity index of the mandibular incisors [31], as it should be emphasised that the grade 4 of ankyloglossia may result from the additional burden on patients, a priori affecting their morphology [37], without the impact of the tongue on the growing mandible. This is why the results of the studies by Yoon et al. [28] and Ardekani et al. [29] are slightly deceiving due to either missing analysis of certain parameter changes depending on the severity of ankyloglossia, or to the involvement of patients with a complete tongue-tie.

Eventually, the current systematic review provides at best a tentative and conditional link of ankyloglossia with the lower face morphology, which in turn emphasises the importance of the studied issue, especially because the number of patients with a severe retrognathic position of the mandible and a medical history of early lingual frenotomy have recently alarmingly increased. To overcome the evidentiary deficiency, a study design aimed at determining the impact of the tongue frenulum length on the lower face morphology should allow for the assessment of skeletal and occlusal parameters in patients representing the entire population, and their comparison with patients with diverse severity of ankyloglossia measured with a validated method, and in a homogeneous control group with normal tongue frenulum length; the study by Srinivasan et al. [27] or Yoon et al. [28] may be the scaffolds for designing ideal methodological models.

The main limitation of our systematic review is the composition and number of participants. Although Jang et al. [26], Yoon et al. [28], Sepet et al. [30], as well as Vaz et al. [31], in contrast to Meenakshi et al. [25], Srinivasan et al. [27], and Ardekani et al. [29], examined large groups of patients, only the studies by Sepet et al. [30] and Vaz et al. [31] involved patients representing the whole population; however, the study by Vaz et al. [31] obtained a lower score due to the absence of both information about the patients’ medical anamneses and characteristics of previous orthodontic interventions.

The lack of control groups in the analysed papers is another limitation of this review. Studies including control groups, the presence of which is an important factor for the reliability of clinical trials, were presented only by Srinivasan et al. [27] and Ardekani et al. [29]. In doing so, Srinivansan et al. [27] pointed out that parameters such as the maxillary and the mandibular intercanine width, as well as the maxillary intermolar width, are statistically significantly smaller in patients with ankyloglossia than in those without this disorder, whereas they are comparable in patients with ankyloglossia. On the other hand, the results reported by Ardekani et al. [29] allowed for erroneous conclusions as to the correlations of malocclusions with ankyloglossia, because by assigning the patients with various malocclusions (Class II and “long face”) to the same group, the authors a priori excluded the possibility of unambiguously considering such material as homogeneous. Consequently, the evidence of a link between ankyloglossia and the hyoid bone position that is related to the mandible morphology and function is weakened.

Summing up, our systematic review provides a very important signpost for fulfilling the serious scientific gap, namely the lack of CCTs and RCTs, without which we are still confused, although at a higher level.

## 5. Conclusions

Overall low evidence quality of the papers included in this systematic review dictates that we should treat the results cautiously. They demonstrated that the increasing severity of ankyloglossia on one hand inhibits maxillary transverse growth and on the other promotes mandibular development, except for the complete tongue-tie. The latter seems to hamper the growing of the mandible; however, the physiological elongation of the lingual frenulum with age clearly opposes the inclusion of grade 4 of ankyloglossia in the list of indications for surgery, unless a pathology is life-jeopardising. Moreover, due to the unclear effect of ankyloglossia on the hyoid bone position, and thus on the possible development of respiratory disorders, more adequate and accurately designed studies are needed.

Notwithstanding the fact that the evidence strength of the articles included in the current systematic review did not allow for statistically significant conclusions to be drawn, it conditionally negated both of the null hypotheses for the following reasons:

1. A shortened lingual frenulum may be one of the factors contributing to maxillary constriction, Class III malocclusion, and mandibular incisor crowding;
2. The patient’s age is relevant when it comes to frenotomy timing, as it differently contributes to clinical benefits in cases of the narrowed maxilla and Class II malocclusion. Specifically, a frenotomy at four years of age in patients with maxillary narrowing allows clinicians to take advantage of the positive tongue effect on the transverse development of the maxilla but poses the risk of the sagittal underdevelopment of the mandible. Therefore, in Class II malocclusion, it is advisable to postpone the lingual frenulum cut until the growth spurt is completed, and that the orthodontic expansion of the Mn C W is carried out prior to completion of the patient’s ninth year of age.

## Figures and Tables

**Table 1:** General information and the aims of the study provided in the papers from the current systematic review.

	Meenakashi et al. [25]	Jang et al. [26]	Srinivasan et al. [27]		Yoon et al. [28]	Ardekani et al. [29]		Sepet et al. [30]	Vaz et al. [31]
			Study Group	Control Group		Study Group	Control Group		
Title	Assessment of lingual frenulum lengths in skeletal malocclusion	Relationship between the lingual frenulum and craniofacial morphology in adults	Skeletal and dental characteristics in subjects with ankyloglossia		Ankyloglossia as a risk factor for maxillary hypoplasia and soft palate elongation: a functional–morphological study	Evaluation of hyoid position in children of 7–11 years old with ankyloglossia in lateral cephalograms		Relationship between mandibular incisor irregularity and type of occlusion in ankyloglossia	Lingual frenulum and malocclusion: an overlooked tissue or a minor issue
Type of publication	CS	CS	CCT		CS	CCT		CS	CS
University	Saveetha Dental College, Saveetha University, India	Department of Orthodontics, Gangneung-Wonju National University Dental Hospital, Gangneung, South Korea	Department of Orthodontics, Sri Ramachandra University, Porur, Chennai		UCLA School of Dentistry, Los Angeles, CA, USA	Shahid Sadoughi University of Yazd, Iran		Istanbul University, Department of Paediatric Dentistry, Istanbul, Turkey	Department of Orthodontics and Dentofacial Orthopedics, PMNM Dental College and Hospital, Bagalkot, Karnataka
Year of publication	2014	2011	2013		2017	2016		2019	2015
Population of the patients	Patients of Saveetha Dental College, Saveetha	Patients enrolled for orthodontic treatment at the Department of Orthodontics	Patients referred to the Department of Orthodontics, Faculty of Dental Sciences, Sri Ramachandra University, Chennai, India for orthodontic treatment		Patients evaluated in a private orthodontic practice	Retrospective cross-sectional study conducted in the paediatric ward of a faculty of dentistry		Patients who were scheduled for dental treatment at Istanbul University, Department of Paediatric Dentistry, Istanbul, Turkey	Patients examined for the presence of a tongue-tie
Number of patients	30	150	57	60	302	30	30	80	700
Females n (%)		79	23 (40.35)	28 (40.35)	187 (61.9)			38 (47.5)	
Males n (%)		71	34 (59.65)	32 (46.66)	115 (38.1)			42 (52.5)	
Age (years) Range or mean (SD)	12–16	24.4–25.9	19.02	19.02	18.1 (9.4)	7–11	7–11	9.36 (1.71)	9–17
The study aim	Comparison of the lingual frenulum lengths in patients with Class I, II, or III malocclusion	Searching for any relationship between lingual frenulum length and cephalometric variables	Comparison of the mean dental and skeletal parameter values: 1. in patients with and without ankyloglossia 2. among the study group in various grades of ankyloglossia		Searching for any relationship between ankyloglossia and variables derived from the dental casts and lateral cephalograms, determining maxillary hypoplasia or soft palate elongation	Comparison of cephalometric values, including a hyoid triangle in patients with and without ankyloglossia		Searching for any relationship between ankyloglossia and the severity of lower incisor crowding, measured on dental casts	Comparison of the lingual frenulum lengths in different malocclusions: Angle’s Class I, II, or III, maxillary constriction, crowding, spacing, open bite, deep bite, crossbite

SD, standard deviation.

**Table 2:** Diagnostic approach to ankyloglossia, provided in the articles from the current systematic review, in relation to sagittal skeletal configuration (Class I, II, or III).

		Meenakashi et al. [25]	Jang et al. [26]	Srinivasan et al. [27]		Yoon et al. [28]	Sepet et al. [30]
				Study Group	Control Group		
Measurement taken to diagnose ankyloglossia and its severity		1. Length of the median lingual frenulum 2. Maximum mouth opening reduction	1. Length of the median lingual frenulum 2. Maximum mouth opening reduction	Free tongue length		1. Tongue functioning 2. Free tongue length	Free tongue length
Measurement technique and the tools applied		In maximum mouth opening and the tongue tip leaned against the incisive papilla, an irreversible hydrocolloid impression was taken for diagnostic casts. The distance between the most anterosuperior point of the lingual frenum and the mesio-incisal edges of the mandibular central incisor was measured in vitro using vernier callipers. This measurement determined the maximum lingual frenulum length	Measuring the median lingual frenulum length in vivo using a lingual frenulum ruler, as proposed by Lee et al. [27]. The amount of maximum mouth opening reduction was measured by using a digital calliper (Digimatic, Mitutoyo, Tokyo, Japan) with a resolution of 0.01 mm and a nominal capacity of 150 mm, as proposed by Marchesan [28]	Kotlow’s method [18] with a digital vernier calliper		1. Assessment of tongue functioning: (a) (QTT) (b) MOTTIP (c) MIO (d) TRMR: MOTTIP divided by MIO 2. Kotlow’s method [18] with a digital vernier calliper	Kotlow’s method [18] with a digital vernier calliper. To reduce the measurement error by hand pressure to the lingual frenulum, the length was recorded as the calliper touched the soft tissue as lightly as possible. The measurement was triplicated
Kotlow’s grade n (%)	1		12 (21.05)			123 (40.7)	45 (56.3)
	2		37 (64.91)			33 (10.9)	23 (28.8)
	3		8 (14.03)			3 (0.99)	12 (15)
	4					1 (0.33)	
Mean ankyloglossia measurement (mm)				8.32	19.17		
MLFL (mm) mean (SD) related to malocclusion	Class I	3.96 (1.46)	3.3 (2.5) **^**				
	Class II	4.08 (0.96)	3.1 (2.6) **^**				
	Class III	5.27 (1.02)	4.9 (9.2) **^**				
MMOR (mm) mean (SD) related to malocclusion	Class I	14.25 (4.7	15.3 (7.3) **^**				
	Class II	16.36 (3.35)	16.5 (8.7) **^**				
	Class III	20.46 (5.76)	22.2 (9.2) **^**				
TRMR related to MIO grading scale n (%)	1					19 (6.3)	
	2					226 (74.8)	
	3					53 (17.5)	
	4					4 (1.3)	

MLFL, median lingual frenulum length; MMOR, maximum mouth opening reduction measured as a difference in the mouth opening according to the position of the tongue either on the mouth floor or with its tip resting on the incisive papilla; QTT, Quick Tongue-Tie Assessment Tool; MOTTIP, Mouth Opening with the Tongue Tip leaned against the Incisive Papilla; MIO, Maximal Interincisal mouth Opening; TRMR Tongue Range of Motion Ratio defined as the ratio of MOTTIP to MIO; **^** Class I, II, III, skeletal malocclusion diagnosed with the ANB angle values. SD, standard deviation.

**Table 3:** The occlusal parameters evaluated in the articles from the current systematic review; descriptive statistics and percentage distribution of the variables either coexisting with or possibly affected by ankyloglossia.

	Srinivasan et al. [27]		Yoon et al. [28]	Sepet et al. [30]	Vaz et al. [31]
	Study Group	Control Group			
Mx C W (mm) mean (SD)	33.0084 (3.983)	35.4022 (2.937)			
Anterior crossbite (%)					0.88
Mx M W (mm) mean (SD)	42.8074 (4.938)	48.3738 (4.200)			
Posterior crossbite (%)					0.88
Maxillary constriction (%)					46.02
Mn C W (mm) mean (SD)	26.5837 (2.858)	27.9598 (1.621)			
Lower incisor irregularity n (%)				Mild 59 (73.8) Moderate 18 (22.5) Severe 3 (3.8)	
Mn M W (mm) mean (SD)	39.8951 (4.197)	41.0918 (4.275)			
Mx crowding (mm) mean (SD)	3.60 (3.26950)	3.26950 (3.17573)			
Mn crowding (mm) mean (SD)	3.49 (2.36347)	4.40 (2.74916)			
Crowding (%)					40.71
Spacing (%)					21.24
Ratio Mx C W:AL (mm) mean (SD)			3.5 (0.9)		
Ratio Mx M W:AL (mm) mean (SD)			1.2 (0.1)		
Ratio Mn C W:AL (mm) mean (SD)			4.7 (1.5)		
Ratio Mn M W:AL (mm) mean (SD)			1.3 (0.2)		
Palatal slope (°) mean (SD)			35.3 (6.5)		
Anterior open bite (%)					18.58
Deep bite (%)					8.85
Overbite (mm) mean (SD)	2.75 (2.157)	2.98 (1.282)			
Class I n (%)				56 (70)	
Class II n (%)				17 (21.3)	
Class III n (%)				7 (8.8)	
Normal occlusion (%)					3.54

Mx C W, maxillary intercanine width; Mx M W, maxillary intermolar width; Mn C W, mandibular intermolar width; lower incisor irregularity, mandibular incisor crowding measured with Little’s irregularity index; Mn M W, mandibular intermolar width; Mx crowding, maxillary tooth size—arch length discrepancy; Mn crowding, mandibular tooth size—arch length discrepancy; ratio Mx C W:AL, ratio of the Mx C W to the canine arch length (the distance from the line connecting the central incisors to the line connecting the canine cusp tips); ratio Mx M W:AL, ratio of the Mx M W to the molar arch length (the distance from the line connecting the central incisors to the line connecting the 1st molar mesio-lingual cusp tips); ratio Mn C W:AL, ratio of the Mn C W to the canine arch length; ratio Mn M W:AL, ratio of the Mn M W to the molar arch length; palatal slope, an angle (in a vertical cross-section of the dental casts) between the line passing through the palatal gingival margins of the 1st molars and the line passing through one of these margins and the deepest point of the palatal vault; SD, standard deviation.

**Table 4:** The maxillofacial morphology parameters evaluated in the articles from the current systematic review; descriptive statistics, percentage distribution, and statistically significant correlation coefficients of the variables either coexisting with or possibly affected by ankyloglossia.

	Meenakashi et al. [25]	Jang et al. [26]		Srinivasan et al. [27]		Yoon et al. [28]	Ardekani et al. [29]	
		MLFL	MMOR	Study Group	Control Group		Study Group	Control Group
Po-Or/ML angle (°) mean (SD)				26.75 (4.563)	25.74 (3.070)			
SN/ML (°) mean (SD)				31.84 (4.225)	31.08 (3.758)			
SN/Me-Go’ (°) Mean (SD)						36.0 (6.1)		
C3-RGN (mm) mean (SD)							66.23 (4.56)	68.56 (5.41)
H-RGN (mm) mean (SD)							33.43 (3.35)	36.83 (2.33)
H-ML (mm) mean (SD)						12.7 (5.2)		
C3-H (mm) mean (SD)							30.90 (2.60)	34.13 (3.69)
H-P (mm) mean (SD)							3.20 (2.13)	7.45 (1.26)
ANB angle (°)		−0.218 **	−0.255 **					
WITS appraisal (mm)		−0.176 *	−0.217 **					
Class I n (%)	10	50 **^**		40 (70.16)				
Class II n (%)	10	50 **^**		15 (26.31)				
Class III n (%)	10	50 **^**		2 (3.5)				
PNS-P (mm) mean (SD)						31.6 (5.0)		
APDI		0.174 *	0.151					
L1-NB distance (mm)		−0.218 **	−0.100					
Interincisal angle (°)		0.260 **	0.175 *					
SNB angle (°)		0.196 *	0.214 **					
Co-Pg distance (mm)		0.196 *	0.348 **					

Po-Or, the Frankfurt plane; ML, mandibular (Go-Gn) line; SN, sella–nasion line; Go’, the transection point of the tangent to the mandibular ramus with the line passing through Me and the highest point of the *incisura premasseterica*; C3-RGN, the distance from the most anteroinferior point of the third cervical vertebra (C3) to the most posterior point of the mandibular symphysis (RGN); H-RGN, the distance from the most anterior and superior point of the hyoid bone (H) to RGN; C3-H, the distance from C3 to H; HP, the height of the hyoid triangle formed by C3, RGN, and H; **^** Class I, II, and III skeletal malocclusions diagnosed with the ANB angle values; PNS-P, the segment drawn from the posterior nasal spina to the lowest point on the uvula tip; APDI, mathematical sum of the facial angle (N-Pog/Po-Or); L1, the tip of the lower central incisor; NB, Nasion-B point line; Co, condylion; Pg, pogonion; SD, standard deviation; r, Spearman correlation coefficient; * *p* < 0.05; ** *p* < 0.01.

**Table 5:** Type of ankyloglossia measurement among studies.

Meenakashi et al. [25]	Jang et al. [26]	Srinivasan et al. [27]	Yoon et al. [28]	Ardekani et al. [29]	Sepet et al. [30]	Vaz et. al. [31]
		Kotlov scale	Kotlov scale	Kotlov scale	Kotlov scale	Kotlov scale
Median lingual frenal length ^L^	Median lingual frenal length					
Maximum mouth opening reduction						
			Tongue Range of Motion Ratio (TRMR)			

^L^: Measurment were made on the prediagnostic casts—risk of error resulting from pressure on soft tissue during the impressions.

**Table 6:** Results of statistical analysis of the skeletal growth pattern in relation to median lingual frenulum length (MLFL) and maximum mouth opening reduction (MMOR).

		Class I	Class II	Class III	Class I-II *p* Value	Class II-III *p* Value	Class I-III *p* Value
Meenakshi et al. [25]	MLFL number of patients mean (SD)	n = 10 3.96 (1.46)	n = 10 4.08 (0.96)	n = 10 5.27 (1.02)	>0.05	>0.05	**
	MMOR number of patients mean (SD)	n = 10 14.25 (4.7)	n = 10 16.36 (3.35)	n = 10 20.46 (5.76)	>0.05	>0.05	>0.05
Jang et al. [26]	MLFL number of patients mean (SD)	n = 50 3.3 (2.5)	n = 50 3.1 (2.6)	n = 50 4.9 (3.0)	>0.05	**	*
	MMOR number of patients mean (SD)	n = 50 15.3 (7.3)	n = 50 16.5 (8.7)	n = 50 22.2 (9.2)	>0.05	**	**

SD, standard deviation; * *p* < 0.05; ** *p* < 0.01.

**Table 7:** Results of statistical analysis of the maxillofacial morphology and occlusion dependent on severity of ankyloglossia in the paper by Skrinivasan et al.

Studied Parameter	Grade 1 Mean (SD)	Grade 2 Mean (SD)	Grade 3 Mean (SD)	Grades Comparison 1/2 *p* Value	Grades Comparison 2/3 *p* Value	Grades Comparison 1/3 *p* Value	Group 1 Mean (SD)	Group 2 Mean (SD)	IT *p* Value
Go-Gn-SN (°)	32.38 (3.114)	32.73 (5.042)	28.75 (4.634)	>0.05	− *	>0.05	31.84 (4.225)	31.08 (3.758)	>0.05
FMA (°)	26.13 (3.399)	27.51 (4.181)	24.83 (4.345)	>0.05	>0.05	>0.05	26.75 (4.563)	25.74 (3.70)	>0.05
Mx C W (mm)	34.6013 (3.9882)	32.6724 (3.1512)	32.9825 (4.4118)	>0.05	>0.05	>0.05	33.0084 (3.983)	35.4022 (2.937)	**−** *
Mn C W (mm)	26.1613 (2.29260)	26.8103 (2.26657)	26.1667 (2.79068)	>0.05	>0.05	>0.05	26.5837 (2.858)	27.9598 (1.621)	**−** *
Mx M W (mm)	43.2563 (3.699)	43.0678 (2.30516)	41.7050 (2.01913)	>0.05	>0.05	>0.05	42.8074 (4.938)	48.3738 (4.200)	**−** *
Mn M W (mm)	38.5275 (3.92119)	40.3730 (3.98773)	39.3333 (3.79793)	>0.05	>0.05	>0.05	39.8951 (4.197)	41.0918 (4.275)	>0.05
Mx crowding (mm)	1.88 (4.155)	3.57 (3.637)	4.83 (1.899)	>0.05	>0.05	>0.05	3.60 (3.26950)	5.05 (3.17573)	**−** *
Mn crowding (mm)	2.50 (2.000)	3.49 (3.288)	4.17 (2.480)	>0.05	>0.05	>0.05	3.49 (2.36347)	4.40 (2.74916)	>0.05
Overbite (mm)	2.13 (1.642)	2.26 (2.127)	4.67 (1.435)	>0.05	**+** *	**+** *	2.75 (2.157)	2.98 (1.282)	>0.05

IT, independent *t* test for means comparison between Groups 1 and 2; **+**, increase; **−** decrease; * statistically significant.

**Table 8:** Results of the statistical analysis of the maxillofacial morphology and occlusion dependent on severity of ankyloglossia diagnosed with two approaches, Tongue Range of Motion Ratio (TRMR) and Kotlow’s method, in the paper by Yoon et al.

	Normal	Grade 1		Grade 2		Grade 3		Grade 4		*p* Value	
	Kotlow Mean (SD)	TRMR Mean (SD)	Kotlow Mean (SD)	TRMR Mean (SD)	Kotlow Mean (SD)	TRMR Mean (SD)	Kotlow Mean (SD)	TRMR Mean (SD)	Kotlow Mean	TMRM	Kotlow
Ratio Mx C W:AL (mm)	03.7 (0.9)	3.6 (0.6)	3.4 (0.8)	3.6 (0.9)	3.0 (0.6)	3.1 (0.7)	2.8 (0.5)	3.0 (0.5)	3.4	**−** **	**−** †
Ratio Mx M W:AL (mm)	1.2 (0.1)	1.2 (0.1)	1.2 (0.1)	1.2 (0.1)	1.1 (0.1)	1.1 (0.1)	1.2 (0.1)	1.1 (0.1)	1.1	**−** **	**−** *
Ratio Mn C W:AL (mm)	4.7 (1.4)	4.9 (1.2)	4.8 (1.6)	4.8 (1.6)	4.2 (1.3)	4.4 (1.2)	4.8 (1.0)	4.9 (0.8)	5.0	>0.05	>0.05
Ratio Mn M W:AL (mm)	1.3 (0.1)	1.3 (0.1)	1.2 (0.2)	1.2 (0.1)	1.2 (0.2)	1.3 (0.2)	1.3 (0.2)	1.4 (0.2)	1.5	>0.05	>0.05
Palatal slope (mm)	35.0 (6.1)	32.4 (6.5)	35.1 (6.5)	35.1 (6.2)	36.5 (8.1)	36.9 (7.5)	42.1 (1.1)	41.4 (1.7)	39.2	**+** **	>0.05
SN-Mn (°)	36.4 (6.0)	36.9 (7.8)	35.5 (6.4)	35.7 (5.8)	35.1 (5.1)	36.6 (6.4)	43.8 (4.0)	40.8 (7.0)	31.5	>0.05	>0.05
H-Mn line (mm)	12.6 (5.1)	13.4 (7.5)	12.5 (5.1)	12.4 (4.8)	13.5 (5.5)	13.3 (5.7)	14.5 (5.8)	15.7 (5.4)	19.4	>0.05	>0.05
PNS-P line (mm)	30.9 (4.7)	30.3 (6.2)	31.5 (5.0)	31.3 (4.6)	34.9 (5.3)	33.5 (5.6)	33.3 (2.9)	34.1 (2.8)	36.3	**+** *	**+** †

SN-Mn angle, the angle between sella–nasion and mandibular (gonion–menton) lines; H-Mn, perpendicular distance from the most anterior and superior point of the hyoid bone to mandibular line; PNS-P line, distance from the posterior nasal spine to the lowest point on the uvula; **+** increase, **−** decrease, * *p* < 0.05; ** *p* < 0.01 on univariate analysis; † *p* < 0.01 on multivariate analysis with a Standard Least Squares Regression Model.

**Table 9:** Results of correlation analysis (r-Pearson coefficient) between MLFL or MMOR and the maxillofacial morphology parameters.

Jang et al. [26]	Ankyloglossia Measurement	SNB (°)	ANB (°)	WITS (mm)	Co-Pg (mm)	APDI	L1-NB (mm)	Interincisal Angle (°)
	MLFL	**+** *	**−** *	**−** *	**+** *	**+** *	**−** **	**+** **
	MMOR	**+** **	**−** **	**−** **	**+** **	>0.05	>0.05	**+** *

* *p* < 0.05; ** *p* < 0.01; **+** increase, **−** decrease.

**Table 10:** The results of the critical appraisal of the CCTs and CSs.

Question from the Checklist	Srinivasan et al. [27]	Ardekani et al. [29]	Meenakshi et al. [25]	Jang et al. [26]	Yoon et al. [28]	Sepet et al. [27]	Vaz et al. [31]
	CCTs	CCTs	CSs	CSs	CSs	CSs	CSs
Were the groups comparable other than the presence of disease in cases or the absence of disease in controls?	Yes	Yes					
Were cases and controls matched appropriately?	Yes	Yes					
Were the inclusion and exclusion criteria defined for both groups?	Yes	No					
Were the same criteria used for the identification of cases and controls?	Yes	Yes					
Were the study subjects and the setting described in detail?			Yes	Yes	Yes	Yes	Yes
Were the criteria for inclusion in the sample clearly defined?			Yes	Yes	Yes	Yes	No
Was the ankyloglossia measured in a standard, valid, and reliable way?	Yes	No	No	Yes	Yes	Yes	Yes
Were the outcomes measured in a valid and reliable way?	Yes	Yes	Yes	Yes	Yes	Yes	Yes
Were confounding factors identified?	Yes	No	No	Yes	Yes	Yes	No
Were the strategies to deal with confounding factors stated?	Yes	No	No	Yes	Yes	Yes	No
Was the appropriate statistical analysis used?	Yes	Yes	Yes	Yes	Yes	Yes	Yes
Assessed risk of bias	Low	High	Moderate	Low	Low	Low	Moderate

**Table 11:** Assessment of the strength of the evidence for the effect of ankyloglossia on the investigated parameters.

Parameter and ESR	ESR	EA
Mx C W	Medium	−
Mx M W	Medium	−
Posterior crossbite	Very low	+
Maxillary constriction	Low	+
Mn C W	Low	−/IR
Lower incisor irregularity	Low	−/Q
Mx crowding	Low	+
Palatal slope	Low	+
Anterior open bite	Very low	+
Deep bite	Very low	+
Overbite	Low	+
Class III	Medium	+/IR
SNB	Low	+
ANB	Low	−
WITS	Low	−
Co-Pg	Low	+
Go-Gn-SN	Low	−
PNS-P	Low	+
APDI	Low	+
L1-NB	Low	+
Interincisal angle (°)	Low	+
Respiratory disorders determined based on unfavourable changes in the hyoid bone and related parameters (C3RGN, HRGN, C3H, HP)	Very low	+/−

ESR—evidence strength of the result according to GRADE evaluation; EA, confirmation of the effect of ankyloglossia on the parameter analysed; −, decrease; +, increase; IR, irrelevant; Q, questionable.
